# Substance Use Disorders in Adolescents: A Qualitative Systematic Review

**DOI:** 10.1111/jan.16967

**Published:** 2025-04-19

**Authors:** Hilal Kara, Dudu Karakaya

**Affiliations:** ^1^ Antalya City Hospital Psychiatric Clinic Antalya Turkey; ^2^ Department of Psychiatric Nursing Akdeniz University Faculty of Nursing Akdeniz University Faculty of Nursing Antalya Turkey

**Keywords:** adolescents, life experience, meta‐synthesis, nursing, substance use disorder

## Abstract

**Introduction:**

It is important for the treatment and care of adolescents using substances to bring out their life experiences relating to substance use.

**Aim:**

To analyse and synthesise qualitative evidence on substance use‐related life experiences of adolescents with substance use disorder.

**Design:**

A systematic review of qualitative studies.

**Methods:**

Systematic searches on the databases PubMed, Science Direct, Scopus, EBSCO CINAHL Complete and Web of Science were conducted between December 2023 and February 2024 and supplemented by manual search strategies. As a result of the search, 1271 articles were accessed and 11 articles were included in the study. In order to assess quality, the critical appraisal skills programme was used. Synthesis of the data was performed using the thematic analysis method recommended by Thomas and Harden (2008). In study reporting, the preferred reporting items for systematic reviews and meta‐analyses (PRISMA) 2020 checklist and enhancing transparency in reporting the synthesis of qualitative research (ENTREQ) guidelines were used.

**Results:**

From 11 included publications, four main themes emerged: starting and continuing substance use, duration of dependence and related factors, the results of substance use and experiences of the treatment process.

**Conclusions:**

Substance use disorder had serious negative effects on the adolescents themselves, their families and their environments. In line with these negative experiences, adolescents made suggestions regarding the substance use disorder treatment process.

**Implications for the Profession and/or Patient Care:**

Professionals working with adolescents can plan their interventions in line with the experiences and suggestions of adolescents who use substances. In addition, governments can take into account the experiences and suggestions of adolescents with substance use disorder when structuring health policies.

**Patient or Public Contribution:**

No patient or public contribution.

**Registration:**

This meta‐synthesis was registered with PROSPERO (CRD42022298218).

**Summary**

- Substance use negatively affects the lives of adolescents.
- Adolescents who use substances are aware of the problems they experience due to substance use and the solutions.
- Professionals working with adolescents can structure their interventions within the framework of the recommendations of adolescents who use substances. Qualitative studies, including the views of adolescents with substance use disorders on the prevention of substance use, can be increased.

## 1 Introduction

Substance use disorder (SUD) continues to be a problem that threatens public health and increases the worldwide disease load (Shen et al. 2023). Results from the Global Burden of Disease drug use disorder have increased by 32.3% in the past 30 years (Shao et al. 2023). Adolescents are more vulnerable to substance use (UNODC 2023). In many regions, such as Oceania, Europe and Asia, adolescents use more substances than the general population (UNODC 2023; UNODC 2024), and the rate of use of new psychoactive substances among schoolchildren is increasing (UNODC 2023). It was reported in a study on the global burden of disease that the disease burden relating to substance use comes down as far as 15‐year‐olds (Shen et al. 2023). The World Health Organisation defines the age of 10–19 as adolescence (WHO n.d.). Due to its developmental characteristics, adolescence is characterised by curiosity, having different experiences, risky behaviour and the importance of a group of friends (Ögel 2018). This increases the risk of adolescents starting to use substances and acquiring SUD (Khalil and Hamdan‐Mansour 2019; Ögel 2018).

Substance use has a negative effect on the lives of adolescents. In individuals who start substance use in adolescence, the risk of substance dependence is high and the negative effects of the substance are greater (UNODC 2023). In systematic review and meta‐analysis studies, it has been found that substance use is related to mental problems such as depression and anxiety (Esmaeelzadeh et al. 2018) and psychosis (Matheson et al. 2023); it opens the way to biological problems such as sleep disorders (Kwon et al. 2019) and eating disorders (Devoe et al. 2021) and causes social problems such as social isolation and loneliness (Ingram et al. 2020). In individuals who start substance use in adolescence, the risk of depression, suicidal behaviour (Gobbi et al. 2019), anxiety disorders (Lowe et al. 2024) and schizophrenia (Godin and Shehata 2022) increases in later life. Implementing intervention programmes in order, for example, to identify substance‐using adolescents in the early stages and to develop awareness, emotion regulation and coping skills is important for preventing biological, psychological and social problems which may be experienced (UNODC‐WHO 2020). In the sustainable development targets for 2030 published by the United Nations, strengthening the prevention and treatment of substance use was targeted (United Nations 2015). Similarly, standards to limit the use of drugs in the world were published by the United Nations Office on Drugs and Crime (UNODC) and the World Health Organisation (WHO) (UNODC‐WHO 2018).

When meta‐synthesis studies on the experiences of individuals with SUD are examined, it is seen that they focus on the treatment experiences of women (Hines 2013) and pregnant women (Tsuda‐McCaie and Kotera 2022), the recovery experiences of individuals recovering from alcohol addiction (Kim et al. 2021) and the substance use experiences of individuals with mental problems (Chorlton and Smith 2016). In the literature, no meta‐synthesis study was found that focused on the experiences of adolescents with SUD in the course of their use of substances. It is thought that analysing studies researching in depth the substance use‐related life experiences of adolescents with SUD can be effective in providing effective care to adolescents and in reducing stigmatisation.

## 2 The Review

### 2.1 Aim

This meta‐synthesis aims to analyse and synthesise qualitative evidence regarding the substance use‐related life experiences of adolescents with SUD. The following research question guided this review: ‘What are the substance use‐related life experiences of adolescents with SUD?’

## 3 Methods

### 3.1 Study Design

Meta‐synthesis is a method that synthesises and interprets evidence from qualitative research to provide an understanding of an event or experience in order to generate new findings from the existing body of research in the literature (Sandelowski and Barroso 2007). The steps proposed by Sandelowski and Barroso (2007) were followed in conducting this meta‐synthesis: (1) identification of the study topic, (2) formulation of the research question and systematic review of the literature, (3) selection and evaluation of studies that meet the inclusion criteria, (4) extraction of data and (5) analysis of data and presentation of results. In study reporting, the preferred reporting items for systematic reviews and meta‐analyses (PRISMA) 2020 checklist (File S1; Page et al. 2021) and enhancing transparency in reporting the synthesis of qualitative research (ENTREQ) guidelines were used (File S2; Tong et al. 2012).

### 3.2 Eligibility Criteria

Inclusion and exclusion criteria were determined by the PICOS method: patient population (P), interventions (I), comparison group (C), outcome (O) and study design (S) (Higgins et al. 2023). There was no intervention or comparison group in this meta‐synthesis, and there was no restriction on the year of publication.

Population (P): Adolescents aged 10–19 with a history of substance use.

Outcome (O): Studies aimed at determining the substance use‐related life experiences in using substances of adolescents with SUD.

Study design (S): Descriptive studies with a qualitative or mixed design published in English.

### 3.3 Exclusion Criteria

Studies with individuals with mental health problems other than SUD or with healthy individuals, studies conducted with those outside the 10‐ to 19‐year age group, grey literature, theses and book sections, nondescriptive experimental studies, quantitative studies and articles published in languages other than English were excluded from the meta‐synthesis.

### 3.4 Search Methods

Comprehensive systematic searches were performed in the databases PubMed, Science Direct, Scopus, EBSCO CINAHL Complete and Web of Science between December 2023 and February 2024. A manual search was performed in the reference lists of the included studies to identify articles that were not retrieved from the databases. The search was conducted independently by two authors (first author and second author). The search was structured using the Boolean operators AND and OR and with descriptors and keywords related to the research question.

Table 1 gives an overview of the keywords; File S3 gives an overview of the details of the search strategy.

**TABLE 1:** Key words.

**Population (P):**	Substance‐Related Disorders, Chemical Dependence, Drug Abuse, Drug Addiction, Drug Dependence, Drug Habituation, Drug Use Disorder, Drug Use Disorders, Prescription Drug Abuse, Substance Abuse, Substance Addiction, Substance Dependence, Substance Use Substance Use Disorder **AND** Adolescents, adolescence, Teenagers, Teens, Youth
**Outcome (O):**	Life Experience, Perception
**Study Design (S):**	Qualitative Research, qualitative study, descriptive qualitative

### 3.5 Study Selection

**FIGURE 1:**
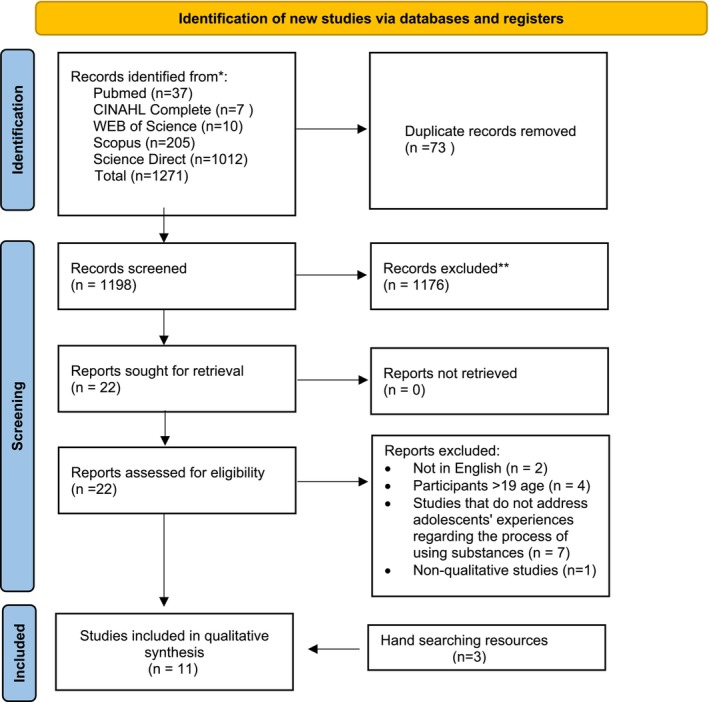
PRISMA 2020 flowchart diagram (Page et al. 2021).

In selecting studies to be taken into the meta‐synthesis, PRISMA guidelines were followed (Page et al. 2021). As a result of the systematic literature search, 1271 articles were accessed. The articles so obtained were transferred to EndNote 20, and after duplications were removed, 1198 studies remained. In order to determine articles for exclusion, each author independently reviewed all studies according to their headings, keywords and abstracts. After that, all of the remaining articles were independently examined by the two authors, and they were assessed for inclusion and exclusion criteria. In this process, the articles were classified as ‘include’, ‘exclude’ or ‘not sure’. Then, the studies were reviewed by the authors together, and eight studies were determined to fit the inclusion criteria. The reference lists of these eight studies were then reviewed, and a further three studies were accessed by a manual search so that the meta‐synthesis was performed with 11 studies. Final decisions on included studies were made when all authors reached an agreement. The process of systematically searching the studies is given in Figure 1 using the PRISMA flowchart (Page et al. 2021).

### 3.6 Quality Appraisal

The methodological quality of the included studies was assessed independently by the two authors, and any inconsistency was resolved by discussion between them. In order to assess the quality of the 11 studies whose coverage was accepted as suitable for the meta‐synthesis, the critical appraisal skills programme (CASP) was used (CASP 2018). The prejudice of the 11 articles studied was found to be low, and they were included in the meta‐synthesis. Table 2 provides information about the quality assessment results of the articles.

**TABLE 2:** Quality assessment of included studies (*n* = 11).

Study	Clear statement of the aims of the research	Appropriateness of qualitative methodology	Research design consistent with the aims of the research	Appropriateness of sampling strategies	Data collection strategy appropriate	Relationship considered between research and participants	Ethical issues considered	Data analysis rigorous	Clear statement of the results	Value of the research
Braciszewski et al. (2018)	Y	Y	Y	Y	Y	Y	Y	Y	Y	Y
Comiskey et al. (2020)	Y	Y	Y	Y	Y	Y	Y	Y	Y	Y
Dallas et al. (2011)	Y	Y	Y	Y	Y	Y	Y	Y	Y	Y
Friese (2017)	Y	Y	Y	Y	Y	Y	Y	Y	Y	Y
James et al. (2019)	Y	Y	Y	Y	Y	Y	Y	Y	Y	Y
Kara et al. (2023)	Y	Y	Y	Y	Y	Y	Y	Y	Y	Y
Malhotra et al. (2007)	Y	Y	Y	Y	Y	Y	Y	CT	Y	Y
Myers et al. (2019)	Y	Y	Y	Y	Y	Y	Y	Y	Y	Y
Pereira et al. (2023)	Y	Y	Y	Y	Y	Y	Y	Y	Y	Y
Vasters and Pillon (2011)	Y	Y	Y	Y	Y	Y	Y	CT	Y	Y
Vázquez et al. (2018)	Y	Y	Y	Y	Y	Y	Y	Y	Y	Y

*Note:* CASP checklist: 1. Was there a clear statement of the aims of the research? 2. Is a qualitative methodology appropriate? 3. Was the research design appropriate to address the aims of the research? 4. Was the recruitment strategy appropriate to the aims of the research? 5. Was the data collected in a way that addressed the research issue? 6. Has the relationship between the researcher and participants been adequately considered? 7. Have ethical issues been taken into consideration? 8. Was the data analysis sufficiently rigorous? 9. Is there a clear statement of findings? 10. How valuable is the research? Appraisal results: Yes (Y), Can't Tell (CT) and No (N).

### 3.7 Data Extraction

Each author independently extracted the data of the included studies, after which agreement was reached on the extracted data. The following information was used to extract data: (1) the author, year and country of the study, (2) the aim of the study, (3) the size of the sample, (4) the study design, (5) the data collection method, (6) the analysis method used and (7) the main themes.

### 3.8 Data Analysis

In the synthesis of data, the thematic analysis method recommended by Thomas and Harden (2008) was used. In the first stage, the two authors repeatedly read the included articles in order to gain a preliminary understanding. The findings of the qualitative studies were encoded inductively line by line. In the second stage, these codes were grouped by comparing similarities and differences, and descriptive themes were created. Finally, the descriptive themes were checked, and new concepts, understandings or hypotheses were determined. At this stage, analytical themes were created that presented the key findings of the primary studies and provided new perspectives to the field beyond the content of the original studies. In the synthesis of data, the two authors worked independently of each other, and the themes determined were agreed on by discussion. The pro‐packet program NVIVO 10 was used to analyse data.

## 4 Results

### 4.1 Study Characteristics

All of the 11 included studies were of qualitative design. The size of the study samples varied from 4 to 47. They were conducted in various different countries: the United States (*n* = 2), Mexico (*n* = 1), Ireland (*n* = 2), Thailand (*n* = 1), Brazil (*n* = 2), India (*n* = 1), South Africa (*n* = 1) and Turkey (*n* = 1). Table 3 provides information about the characteristics of the included studies.

**TABLE 3:** Characteristics of included studies.

Author/year/country	Aim	Size of the sample	Study design	Data collection method	Analysis method used	Main themes
Braciszewski et al. (2018), USA	To identify ways in which to increase engagement with substance use treatment by understanding preferences for and barriers to services for youth formerly in foster care.	24	Qualitative research	Focus group interview, semi‐structured interview, audio recorded	Thematic analysis	1. Perceptual facilitators and barriers a. Understanding a person's readiness to change b. Feeling judged and controlled c. Needing someone with lived experience 2. Concrete facilitators and barriers a. Building strong social support b. Designing tailored interventions
Comiskey et al. (2020), Ireland	To seek to understand the perceptions and experiences of crime and policing of adolescents who used cannabis before their treatment entry	8	Qualitative research, interpretivist paradigm	One‐to‐one semi‐structured interview, audio recorded	A deductive thematic analysis	1. Early Days—Early crimes, debt and developing violence 2. Later Days—Increasing violence, the oppressed becomes the oppressor 3. Getting Caught—varying consequences, from cautions to custody 4. Family and others 5. Family debt and effects on others 6. Intergenerational drug use
Dallas et al. 2011, Thailand	To understand the challenges male Thai adolescents face as they transition from drug dependence to being drug free.	12	Qualitative research, Heideggerian phenomenology	Audio‐taping, field notes, a reflexive journal	Thematic analysis	1. Being a drug abuser a. It is not what I thought it would be b. It is an undesirable existence c. I lost my place in the world 2. Learning from looking back a. Love and home bring me back b. I struggle to stay clean 3. Constructing a new world a. Look, I am a new person b. This is the life I want
Friese (2017), USA	To examine how teens in an environment where medical marijuana is legal and marijuana has been decriminalised perceive marijuana and their reasons for or against use.	47	Qualitative research	Semi‐structured interviews, audio recordings and individual meeting	Thematic analysis	1. Regular marijuana users 2. Occasional marijuana users 3. Former regular marijuana users 4. Former occasional users 5. Never used marijuana
James et al. (2019), Ireland	To provide an insight into the experiences of teenage cannabis users attending treatment, focusing on their journey into cannabis use, the effect of cannabis on their life and the experience of attending treatment.	8	Qualitative research design, interpretivist paradigm	One‐to‐one interviews, structured and semi‐structured questions, audio‐recorded	Thematic analysis	1. Cannabis initiation and heavy use 2. Cannabis ambivalence 3. Stealing and dealing 4. Treatment 5. Damage to relationships 6. Parental cannabis use
Kara et al. (2023), Turkey	To portray the life experiences of adolescents using psychoactive substances with regard to their substance use.	15	Qualitative study, interpretive paradigm, descriptive phenomenological approach	Digital sound recording device, semi‐structured interview form, individual meeting	Content analysis approach	1. Experiences before substance use a. Life before substance use b. Predisposing factors for substance use 2. Experiences during substance use a. Symptoms of addiction b. Problems caused by substance use c. Family attitude to substance use d. The meaning attached to substances 3. Experiences of the treatment process a. Motivation b. Difficulties experienced during treatment c. The need for support d. Treatment results 4. Prevention recommendations a. For peers b. For families c. For society
Malhotra et al. (2007), India	To study the pattern of drug use, reasons for initiation and the perception of the effects of using drugs, among juveniles in conflict with law	34	Qualitative study	Focus group discussions, The topic outline guide, field notes	Thematic analysis	1. Drug use 2. Initiation into drugs 3. Drug availability 4. Perception of effects of drug use
Myers et al. (2019), South Africa	To explore adolescents' perceptions of effective substance use disorder treatment and possible barriers to completing PROMs and PREMs to guide efforts to adapt the South African Addiction Treatment Services Assessment (SAATSA) for adolescents.	38	Qualitative research	Semi‐structured discussion guide, focus group discussions, audio recorded	Framework Method	1. Treatment goals and priorities 2. Perceptions of SUD service needs 3. Treatment experiences and engagement in care 4. Views of patient‐reported outcome and experience measures
Pereira et al. (2023), Brazil	To identify the experiences and challenges during psychiatric hospitalisation from the perception of adolescent users of psychoactive substances (PSA)	4	Exploratory, qualitative study	Semi‐structured interviews, mobile phone and a portable recorder, individually	Content analysis method	1. Trajectory followed until the current care 2. Experiences and challenges faced during hospitalisation
Vasters and Pillon (2011), Brazil	To get a brief picture of adolescents' trajectory in specialised drug use treatment, in the attempt to identify their general characteristics, drug use‐related aspects, how these adolescents reached specialised treatment and how they perceive the factors that facilitate or hamper their continuation in treatment	14	Qualitative research	Semi‐structured interview	Thematic analysis	1. Motives for continuous drug use 2. Desire or intensity of use 3. Motives for changes in drug consumption patterns 4. How he reached specialised treatment 5. Factors helping to continue treatment or reduce consumption 6. Factors hampering treatment 7. Contributions to treatment 8. Characteristics of an attractive/effective service
Vázquez et al. (2018), Mexico	To understand the life experience of adolescents who use illicit drugs	11	Qualitative research, phenomenological approach based on Martin Heidegger's assumptions	Phenomenological interview	Unstated	1. Family roughness 2. Experiencing the world of drug consumption 3. Hope ‘To Be There’

### 4.2 Qualitative Synthesis

Four main themes emerged from this meta‐synthesis: starting and continuing substance use, duration of dependence and related factors, results of substance use and experiences of the treatment process (Table 4).

**TABLE 4:** Themes and subthemes.

Themes	Sub‐Themes	Braciszewski et al. (2018)	Comiskey et al. (2020)	Dallas et al. (2011)	Friese (2017)	James et al. (2019)	Kara et al. (2023)	Malhotra et al. (2007)	Myers et al. (2019)	Pereira et al. (2023)	Vasters and Pillon (2011)	Vázquez et al. (2018)
Starting and continuation of substance use	Individual factors			**✓**	**✓**		**✓**	**✓**		**✓**	**✓**	
	Family factors	**✓**	**✓**		**✓**	**✓**	**✓**			**✓**	**✓**	**✓**
	Environmental factors		**✓**		**✓**	**✓**	**✓**	**✓**		**✓**	**✓**	
Duration of dependence and related factors	Symptoms of dependence			**✓**	**✓**	**✓**	**✓**	**✓**				**✓**
	Meaning attached to the substance			**✓**	**✓**	**✓**	**✓**					
Results of substance use	Individual consequences		**✓**	**✓**	**✓**	**✓**	**✓**	**✓**	**✓**		**✓**	**✓**
	Family consequences		**✓**	**✓**		**✓**	**✓**	**✓**	**✓**			
	Social consequences	**✓**		**✓**		**✓**	**✓**	**✓**				
	Legal problems		**✓**	**✓**		**✓**	**✓**	**✓**		**✓**		**✓**
Experiences of the treatment process	Applying for treatment				**✓**	**✓**	**✓**			**✓**	**✓**	
	Difficulties experienced during treatment						**✓**			**✓**	**✓**	
	Experiences after treatment			**✓**			**✓**		**✓**	**✓**	**✓**	**✓**
	Recommendations regarding treatment	**✓**		**✓**	**✓**	**✓**	**✓**		**✓**	**✓**	**✓**	**✓**

### 4.3 Main Theme 1: Starting and Continuation of Substance Use

#### 4.3.1 Individual Factors

In this subtheme, it was seen that many individual factors affected adolescents with SUD in starting and continuing substance use. The adolescents in the included studies stated that individual factors which affected them in starting substance use were curiosity and desire (Kara et al. 2023; Malhotra et al. 2007; Pereira et al. 2023), unawareness of the addictive effect of the substance (Dallas et al. 2011; Friese 2017), inadequacy of free‐time activities (Kara et al. 2023; Vasters and Pillon 2011), inadequate coping skills (Kara et al. 2023; Malhotra et al. 2007; Vasters and Pillon 2011) and having an aggressive personality and academic problems (Kara et al. 2023).

‘When I got angry, it (the substance) directly came to my mind, because I knew it would calm me – it really calms. Usually when I'm angry I go looking for it’. (Kara et al. 2023)

‘Is marijuana considered a drug?’ (Friese 2017)

#### 4.3.2 Family Factors

It was found that family factors affected adolescents in starting substance use. In the included studies, the adolescents stated that the family factors which affected them in starting substance use were the presence of fighting and violent behaviour (Kara et al. 2023; Pereira et al. 2023; Vázquez et al. 2018), substance use by family members (Comiskey et al. 2020; Friese 2017; James et al. 2019; Kara et al. 2023; Vasters and Pillon 2011; Vázquez et al. 2018), lack of adequate support from the family (Braciszewski et al. 2018; Kara et al. 2023; Vasters and Pillon 2011; Vázquez et al. 2018), separation of the father and mother (Kara et al. 2023; Vasters and Pillon 2011), parents not showing love and affection (Kara et al. 2023; Vázquez et al. 2018) and socioeconomic problems (Kara et al. 2023; Pereira et al. 2023).

‘…as soon as I smelled it, my dad came straight into my head because that smell was always used to associated with him, …, he was smoking joints all the time’. (Comiskey et al. 2020).

‘My dad was in prison… If he'd treated me like his son there'd have been no need for this (substance). I've never seen a father's love in my life so far. It's all reprimands, quarrelling, fighting…’. (Kara et al. 2023)

#### 4.3.3 Environmental Factors

The adolescents in the included studies stated that the environmental factors that affected their starting to use substances were friends (Comiskey et al. 2020; Friese 2017; James et al. 2019; Kara et al. 2023; Malhotra et al. 2007; Pereira et al. 2023; Vasters and Pillon 2011), the media (Malhotra et al. 2007), social activities such as parties (Friese 2017; Vasters and Pillon 2011) and easy access to substances (Kara et al. 2023; Malhotra et al. 2007).

‘We used to take drugs because our friends did so; we started taking them after watching our friends; initially friends offered us and slowly it became a habit’. (Malhotra et al. 2007).

‘I was going out with my friends. They said they were smoking A4 (paper‐impregnated bonsai). I don't want it, I said. They took it out of the packet and gave it. “Have you put something in it?” I said. They said they hadn't. I lit up too. Then my stomach turned, it was my first time. I started vomiting. After that my body got used to it. I started wanting it all the time’. (Kara et al. 2023).

### 4.4 Main Theme 2: Duration of Dependence and Related Factors

#### 4.4.1 Symptoms of Dependence

Adolescents with SUD experience symptoms of dependence. Those included in the studies reported that the symptoms they experienced were a desire to use the substance and an inability to control its use (James et al. 2019; Kara et al. 2023; Malhotra et al. 2007), only thinking about the substance (James et al. 2019; Kara et al. 2023), an inability to quit the substance (Dallas et al. 2011; Kara et al. 2023; Malhotra et al. 2007), tolerance and trying different substances (James et al. 2019; Kara et al. 2023; Vázquez et al. 2018), continuing use despite suffering harm (Friese 2017; James et al. 2019; Kara et al. 2023; Vázquez et al. 2018) and deprivation (James et al. 2019; Kara et al. 2023; Malhotra et al. 2007).

‘(…) I tried crystal, and it was what affected me too much, I did many things I was not supposed to, that's how the drug is, you even feel that you need it to live, as they say, I live to do drugs and I do drugs to live’. (Vázquez et al. 2018).

‘…you just kind of have to realise you are just ruining your own life ‘cos I really was ruining my life like I wouldn't go to school, I would just sit in and put debt on me head and get just stoned. I didn't care about anything except smoking grass…’. (James et al. 2019).

#### 4.4.2 Meaning Attached to the Substance

Adolescents attach different meanings to the substance that they use. The adolescents in the included studies stated that they saw the substance that they used as a friend (Kara et al. 2023), an enjoyable and loved activity (Dallas et al. 2011; Friese 2017), something that solved their problems (James et al. 2019) or an easy way to socialise, and a beautiful time to look back on as they got older (Friese 2017). The adolescents also compared the substance that they used to various objects with negative associations such as a knife, an ice mountain, a trash can, a robot, a weapon or poison (Kara et al. 2023). When the adolescents were talking about the substance in another study, they viewed the drug positively because it was a plant, it was less harmful than other substances, it did not cause dependence and it did not make them do harmful things; it could be used medicinally, attempts had been made to legalise it and it was widely used (Friese 2017).

‘Me and my friends just get together and smoke some weed, and just have a good time […]. I like it because I'm just creating memories and something to look back on when I'm old’. (Friese 2017).

‘I'd compare it to a weapon, because when you've got a weapon in your belt, you're the strongest. When you've got the ingredient (the substance) in your pocket, you're the strongest then too, because people are in need of you. But as you smoke that ingredient, you die. When a gun is brought out and put to your head you die too. It's the same thing’. (Kara et al. 2023).

### 4.5 Main Theme 3: Results of Substance Use

#### 4.5.1 Individual Consequences

Substance use causes many problems in adolescents at an individual level. The adolescents in the included studies spoke of economic effects of substance use such as getting into debt (Comiskey et al. 2020; James et al. 2019; Kara et al. 2023), not being able to get money from the family, inability to work, selling things or doing work that they did not want for the substance and spending a lot of money for the substance (Kara et al. 2023) and effects on physical health (Friese 2017; James et al. 2019; Kara et al. 2023; Malhotra et al. 2007; Myers et al. 2019; Vasters and Pillon 2011; Vázquez et al. 2018) and mental health (Comiskey et al. 2020; Dallas et al. 2011; Friese 2017; James et al. 2019; Kara et al. 2023). The adolescents stated that with substance use, their academic success fell (Kara et al. 2023; Vasters and Pillon 2011), that they had been caught at school under the influence of the substance (Friese 2017) and that they had left school (Vasters and Pillon 2011). Some of the adolescents mentioned that they had experienced individual problems in using substances such as lying to their families and hiding their substance use (Comiskey et al. 2020; James et al. 2019; Kara et al. 2023), and understanding the importance of the family and the place where they lived when they were in prison (Dallas et al. 2011).

‘Like, I just felt like when I was in bed and I wasn't stoned, I couldn't sleep, my mind wouldn't shut off. When I was in bed stoned, I was asleep in two seconds like’. (James et al. 2019).

‘I had hallucinations continually. I looked at a tree knowing it was a tree, but at first I thought there was a man hanging by his neck and he would come to kill me…. Open the window…at my home…over there…over there…I saw him and he haunted me continually. I tried to close my eyes, but I couldn't sleep’. (Dallas et al. 2011).

#### 4.5.2 Family Consequences

Substance use in adolescents affects their families. The adolescents in the included studies stated that substance use brought about family problems such as family quarrels (Comiskey et al. 2020; Kara et al. 2023; Malhotra et al. 2007), changes in their relations with their families (Dallas et al. 2011; Myers et al. 2019) and the family receiving threats because of the debts of the individual who used substances and the child having to pay the debt (Comiskey et al. 2020; James et al. 2019). Also, the adolescents stated that when they learned about their substance use, their families reacted with, for example, worry (James et al. 2019), violence (Dallas et al. 2011; Kara et al. 2023), disappointment (Dallas et al. 2011; Kara et al. 2023) or lack of trust (Comiskey et al. 2020; James et al. 2019; Kara et al. 2023), by handing them over to the police, taking them out of school, throwing them out of the house, not giving them money or discriminating between siblings (Kara et al. 2023). Some of the adolescents reported that when their families found out that they were using substances, they received reactions such as indifference (Comiskey et al. 2020), treating it as normal or not changing (Kara et al. 2023) and attempting to persuade them to give up the substance (Malhotra et al. 2007).

‘My mum's face had a look of disappointment. She asked what was going on. I told her the police arrested me for a positive urine test. She asked me when I used it and slapped my face, one time, then walked into the house. I felt sorry….my mom already forgave me the first time then I repeated the mistake again. I was very sorry’. (Dallas et al. 2011).

‘I always had me Ma and Da fighting because like if I didn't have a joint all the time, I would be snappy and I would be shouting at ya and I would be screaming like and I just wouldn't be me, it's like it turns you into someone you are not’. (Comiskey et al. 2020).

#### 4.5.3 Social Consequences

Substance use affects the social life of adolescents who use substances. The adolescents in the included studies reported that they fought with the people around them when they were using substances and that they experienced problems with those around them, such as a breakdown in communication (Dallas et al. 2011; James et al. 2019; Kara et al. 2023; Malhotra et al. 2007), forming relationships with friends using substances based on benefit (James et al. 2019; Kara et al. 2023) and the end of social life and being in dangerous places (Kara et al. 2023), and that they were stigmatised by those around them (Braciszewski et al. 2018; Dallas et al. 2011; Kara et al. 2023; Malhotra et al. 2007).

‘I lived my life as if I was a robber. I had to avoid others and hide. I even stopped talking to my friends. I was freaking out…just hearing a leaf fall…Crack…I would turn my head left and right looking for someone’. (Dallas et al. 2011).

‘People called us druggies, they did not let us play with their children, they didn't even call us to any party’. (Malhotra et al. 2007).

#### 4.5.4 Legal Problems

Substance use causes legal problems for adolescents. The adolescents in the included studies stated that they had legal problems such as theft (Comiskey et al. 2020; James et al. 2019; Kara et al. 2023; Malhotra et al. 2007; Pereira et al. 2023; Vázquez et al. 2018), selling substances (Comiskey et al. 2020; James et al. 2019; Kara et al. 2023), violence and harassment from friends using substances (Kara et al. 2023; Pereira et al. 2023), legal proceedings against them (Comiskey et al. 2020; Dallas et al. 2011; Kara et al. 2023; Malhotra et al. 2007) and fighting and violence (Kara et al. 2023; Malhotra et al. 2007; Pereira et al. 2023).

‘I was selling drugs at one stage as well…so I wasn't paying for it…. So now that I had the weed when I got money I could buy clothes ‘cos I didn't spend it on weed, so you know what I mean’. (James et al. 2019).

‘(…) when I used marijuana, it calmed me down, then I started to try pills, then hallucinogens, I felt different reactions, when I tried amphetamines, and combined them with pills and marijuana, it pushed me to commit robbery’. (Vázquez et al. 2018).

### 4.6 Main Theme 4: Experiences of the Treatment Process

#### 4.6.1 Applying for Treatment

The adolescents with SUD in the included studies spoke of the ways they had applied for treatment: by themselves (Kara et al. 2023; Pereira et al. 2023; Vasters and Pillon 2011), application by the legal route (Pereira et al. 2023; Vasters and Pillon 2011), application through parents (Kara et al. 2023; Vasters and Pillon 2011) and unwanted hospitalisation (Pereira et al. 2023). They stated that the factors that affected application for treatment were realising the negative effects of the substance (Friese 2017; James et al. 2019; Kara et al. 2023), pressure from people around them (James et al. 2019) and problems at school (Friese 2017).

‘When was the first time? I had the first hospitalization last year. I wanted to. I decided to get the treatment’. (Pereira et al. 2023).

‘In the end my mother said, “I won't forgive you. Either go and rot, or otherwise let's go back to our old life, like human beings.” If it wasn't for my mother, I wouldn't have had any intention of leaving this. I couldn't have left it anyway, I wouldn't have left it… I really came here to escape from it (the substance). So as not to dishonor my mother’. (Kara et al. 2023).

#### 4.6.2 Difficulties Experienced During Treatment

Adolescents applying for treatment for SUD face many problems. The adolescents in the included studies spoke of difficulties experienced relating to problems with access to treatment for those under 18 (Kara et al. 2023; Vasters and Pillon 2011), being away from the family, worry about safety, restriction of freedom, staying in hospital for a long time (Pereira et al. 2023) and the side effects of medication and treatment not doing any good (Kara et al. 2023).

‘The mere fact of being there. Ah, because you're deprived of your freedom […]’. (Pereira et al. 2023)

‘My family applied to the hospital. In Pamukkale University, in Denizli. Because I'm a child, they couldn't treat me there, and they couldn't prescribe the medication to someone under 18. I got no good out of it there, so they applied here. We come here every week’. (Kara et al. 2023)

#### 4.6.3 Experiences After Treatment

The adolescents in the included studies mentioned experiences after treatment for SUD relating to spending time with their families and improving relations with their families (Dallas et al. 2011; Kara et al. 2023; Vasters and Pillon 2011), being able to work, save money and improve their economic situation (Dallas et al. 2011; Kara et al. 2023), bringing their roles in the family into line, being able to continue with school and to complete their schooling (Dallas et al. 2011), being able to focus their thoughts (Pereira et al. 2023), improving sleep, being able to eat and put on weight and to realise that they were living (Kara et al. 2023).

‘I realized I was alive… I left off; a month passed and I made up with my family. There was no peace at home… I went to the industrial area to learn to work, to learn a trade… I immediately bought myself a telephone. I mean, you give it all for that (the substance). When you're not giving it, you start to save up money…’. (Kara et al. 2023)

‘It helped me center my thoughts… because I was very confused. It also made things click for me because… even after freaking out, I still missed the drug. It helped me realize it wasn't good for me’. (Pereira et al. 2023)

The adolescents in the included studies said that after having treatment for SUD, they had future plans for living with their families (Vázquez et al. 2018) and for education and work (Myers et al. 2019; Vázquez et al. 2018).

‘(…) I hope for my life, from now on, to get out of all this, and go back to my family, which always has an open door’. (Vázquez et al. 2018).

‘Finishing school so I can go study at the university’. (Myers et al. 2019).

#### 4.6.4 Recommendations Regarding Treatment

The adolescents with SUD made some suggestions for their peers regarding treatment. The adolescents in the included studies recommended to their peers that they should first want to give up substance use (Braciszewski et al. 2018; Vasters and Pillon 2011); they should see the bad results of the substance (Braciszewski et al. 2018; Pereira et al. 2023); they should have free‐time activities (Pereira et al. 2023; Vasters and Pillon 2011); they should keep to treatment and be decisive about continuing with treatment (Dallas et al. 2011; Kara et al. 2023; Vasters and Pillon 2011; Vázquez et al. 2018); and they should recount what they had experienced (James et al. 2019).

‘If they don't want help, what can you do? You can't help someone who doesn't want help. If he feels as though he's doing fine, there's nothing you can tell him to change his mind. You can sit there and constantly talk and talk and talk, but if he feels as though there's absolutely nothing wrong with the situation, what can you do?’ (Braciszewski et al. 2018).

The adolescents in the included studies recommended to families that they should support their children during treatment (Braciszewski et al. 2018; Kara et al. 2023; Myers et al. 2019; Pereira et al. 2023; Vasters and Pillon 2011).

‘It will help me a lot having my family next to me, knowing that they're with me [in this] and knowing that they're also willing to make some changes as well’. (Myers et al. 2019)

The adolescents recommended changing their circle of friends during treatment (James et al. 2019; Vasters and Pillon 2011), and recommended that friends in their circle should not be judgmental (Braciszewski et al. 2018).

‘Like, say you fall off the tracks… I know what it's like to fall off and get shut out and it's like, “Oh, well, I thought you were supposed to be there for me”. Family and friends, real family and friends, are not supposed to just shut you out for a mistake’. (Braciszewski et al. 2018).

The adolescents made suggestions such as that health professionals should behave in a friendly way and behave appropriately for the patient's age (Myers et al. 2019); they should show interest (Myers et al. 2019; Pereira et al. 2023) and not be judgmental (Braciszewski et al. 2018; James et al. 2019; Myers et al. 2019; Pereira et al. 2023; Vasters and Pillon 2011). Also, the adolescents recommended interventions in which treatment should be interesting and motivating (Myers et al. 2019; Vasters and Pillon 2011), psychosocial counselling services should be given (Myers et al. 2019; Pereira et al. 2023), individual interventions should be made (Braciszewski et al. 2018) and emotional regulation and coping skills should be improved (Myers et al. 2019). They also recommended with regard to the location of the treatment that it should be carried out in a suitable place with their peers (Myers et al. 2019; Vasters and Pillon 2011), and that the activities performed in the treatment should be varied (Myers et al. 2019; Pereira et al. 2023), among their suggestions on the therapeutic environment (Braciszewski et al. 2018; Kara et al. 2023; Myers et al. 2019; Pereira et al. 2023; Vasters and Pillon 2011).

‘They should teach you to use words instead of your fists… how to control your emotions’. (Myers et al. 2019).

‘They could care more. Care more for… especially given what the teenagers are doing there among themselves. Because they did many wrong things […] some nurses turn a blind eye. They'd say, “that kid's a screw‐up,” I've seen, I've witnessed, “go get him, I'll keep watch.” […] The nurses should be more vigilant, more attentive, you know… because there are drugs there, I'm sure of it, there are people being beaten up, people suffering, people wanting to kill themselves’. (Pereira et al. 2023).

## 5 Discussion

In this meta‐synthesis, the substance use experiences of adolescents with SUD are systematically set out in depth. It is seen that the factors affecting starting and continuing substance use in adolescents with SUD and the problems they have with substance use and during treatment are shaped in an individual, family and social framework. This shows that the struggle against SUD can be conducted with a multidisciplinary approach. One important result reached in the meta‐synthesis was that the suggestions relating to the treatment of adolescents with SUD showed similarity with the literature. This shows that they have become aware of the problems of adolescents who use substances and the solutions. It is thought that interventions regarding adolescents with SUD, especially the suggestions of adolescents with those experiences, are important. Adolescents' adherence to intervention programmes can be increased with prevention and treatment programmes that are created within the framework of recommendations of adolescents with SUDs.

This meta‐synthesis shows the factors affecting adolescents' starting and continuing to use substances, perceptions concerning substance use and problems experienced in connection with substance use. It was seen in the meta‐synthesis that individual, family and social factors affected adolescents in starting substance use. In a systematic review study by Nawi et al. (2021), it was reported that individual, family and social risk factors affected adolescents in starting substance use. In the study, it was determined that adolescents' attitudes and perceptions regarding the substance were addressed within the framework of risk factors (Nawi et al. 2021). It was also seen in this meta‐synthesis that adolescents attached positive and negative connotations to the substance. It can be said that the positive connotations of an enjoyable and loved activity or friends are effective in starting and continuing substance use, while meanings with negative connotations such as poison, knife or gun are effective in deciding on treatment. Substance abuse disorders negatively affect an individual's mental health, safety and well‐being (UNODC 2023). The adolescents in the meta‐synthesis who used substances stated that they had been exposed to many negative individual, family, social and legal life events. In systematic reviews and meta‐analysis studies, it has been found that substance use causes physical (Devoe et al. 2021; Kwon et al. 2019), mental (Esmaeelzadeh et al. 2018; Rioux et al. 2021), legal (Tharshini and Ibrahim 2023) and social (Gubbels et al. 2019; Ingram et al. 2020; Kilian et al. 2021; Magnan et al. 2024) problems. Similarly, in a meta‐synthesis by Mardani et al. (2023) with families affected by addiction, the presence in the family of an individual using substances caused many physical, mental, economic and social problems for the family. From these results, it can be said that the problems experienced in connection with the use of substances show great similarity to the factors affecting adolescents in starting and continuing to use substances. In this way, it is thought that a multidisciplinary understanding should be developed in the prevention, treatment and rehabilitation of substance use. Therefore, schools, families and people living in the community should be included in intervention programmes to be conducted for adolescents who use substances. Professionals who work with adolescents, such as teachers, social workers and health professionals, should plan initiatives such as raising awareness for children and adolescents, families and society, strengthening emotion regulation and self‐management skills, increasing self‐esteem, developing life skills, improving parenting and strengthening socialisation (Bulechek et al. 2017). The effectiveness of the initiatives which they plan should be tested in studies with a high level of evidence.

It was seen in this meta‐synthesis that adolescents who decided to be treated entered a process of change, that they met many problems in this process and that as a result of their experiences, they had suggestions relating to the treatment process. In a study by Farhoudian et al. (2022), obstacles to the treatment of SUD were listed as fear, wrong beliefs, stigmatisation, inadequate social support, obstacles to services providing treatment and legal and political hindrances. Factors which made treatment easier were reported as personal motivation, the presence of supportive family and friends and the formation of services providing treatment (Farhoudian et al. 2022). The published standards for SUD treatment state that the treatment of adolescents should address all the personal needs of the adolescent and be structured according to their individual needs, family therapies should be included, a psychosocial treatment approach specific to the child's age and experiences should be adopted, adolescents' hobbies should be included in the treatment process and sports activities should be included. It is stated that it is important to assess the risks to the adolescents undergoing treatment for violence, exploitation, self‐harm and suicide and to identify these problems at an early stage (UNODC‐WHO 2020). It was seen in this meta‐synthesis that published standards on SUD treatment and the adolescents' suggestions on treatment were similar. This shows that even though adolescents with SUD had experienced negative life events, they were aware of the problem and ways to solve it. In this meta‐synthesis, the adolescents stated that in the treatment process, adolescents must first want to be treated, otherwise they will not conform to treatment and it will not be of benefit to them. In addition, adolescents made specific suggestions such as developing coping and emotion regulation skills during the treatment process, creating motivation for change, creating an environment where there is freedom of choice, having more than one option in interventions and not stigmatising and discriminating. In this context, it may be said that governments should set up their health policies relating to the treatment of individuals with SUD, the environments where adolescents are treated and the interventions applied during treatment in conformity with international standards. Health professionals and researchers can develop individual treatment programmes which are specific to children and adolescents. Also, health professionals can avoid showing a stigmatising attitude to adolescents during treatment and can plan training for their colleagues on this topic. The views of adolescents with experiences of SUD treatment can then be taken into account in planning services for adolescents receiving this treatment.

Another result relating to experiences of treatment in this meta‐synthesis is adolescents' plans relating to work, family and school. This shows that the adolescents want to get back the lives they have lost in connection with substance use and that they are hopeful for the future. Hope is an important element in the recovery process (SAMHSA 2012). In a study by Gutierrez et al. (2020), it was stated that hope has a mediating effect on recovery from SUD and that it can help an individual keep away from substance use. SUD treatment is a journey with ups and downs (Ögel 2018). In this process, a positive contribution can be made to the treatment process by supporting substance‐using adolescents with hope. Professionals working with adults can plan interventions to support hope, such as instilling hope in adolescents who use substances (hope inspiration) (Bulechek et al. 2017). Also, it is important for adolescents to gain healthy life skills and behaviours in order to keep away from substances during treatment and rehabilitation. Training can be given to adolescents receiving treatment for SUD on social skills, a profession, job placement, school orientation and life skills. In this way, health professionals, social workers, civil society institutions and administrative units can work together to support the reintegration of children and adolescents into society.

### 5.1 Review Limitations

This meta‐synthesis has limitations. Only articles in English were included in the meta‐synthesis. For this reason, the studies included in the meta‐synthesis may not reflect the experiences of adolescents from different countries and cultures. The sample of this study is limited to the studies included in the meta‐synthesis. Also, studies obtained from countries with low, medium and high incomes were included in this meta‐synthesis. No examination was made of how the life experiences of individuals with SUDs living in these countries differ according to the environment in which they live.

## 6 Conclusion

Many factors affect adolescents with SUD in starting and continuing substance use. During the course of substance use and treatment, they have serious individual, family and social problems. Individual, family and social factors influence them in starting to use substances, and substance use has a negative effect in these areas. Family factors are more prominent than others in starting adolescents in the use of substances, but the negative consequences of substance use mostly affect the adolescents themselves. Also, the adolescents made recommendations for the treatment process regarding their bad experiences, which were in line with international treatment standards. This shows that the adolescents were aware of the problem of substance use and a solution to this problem. In this regard, when intervention plans are being formed with regard to adolescents with SUD, their experiences and suggestions should be taken into account.

## 7 Relevance to Clinical Practice

Professionals working with adolescents can plan individual, family and societal interventions, taking into account the experiences and recommendations of adolescents who use substances. They can provide education to children and adolescents on life skills enhancements, self‐awareness enhancements, resiliency promotion, emotional regulation and development of coping skills. Interventions can be planned with family‐oriented and family‐based programmes to strengthen families and parents. The media, civil society institutions, health institutions and schools can work together to plan interventions to increase social awareness of SUD. Training sessions on this subject can be organised for the community, and campaigns can be conducted against stigmatisation. With regard to society, interventions can be planned to increase societal awareness and reduce stigmatisation. It was seen in the meta‐synthesis that there were a limited number of qualitative studies dealing with suggestions for the prevention of substance use by adolescents with SUD. Thus, qualitative studies on the views and suggestions of adolescents with SUD on the prevention of substance use may be increased. In addition, governments can take into account the experiences and suggestions of adolescents with SUD when structuring health policies.

## Author Contributions

H.K.: conceptualisation, data curation, formal analysis, investigation, methodology, resources, software, validation, visualisation, writing – original draft and writing – review and editing. D.K.: conceptualisation, data curation, formal analysis, investigation, methodology, project administration, resources, software, supervision, validation, visualisation, writing – original draft and writing – review and editing.

## Ethics Statement

Ethical approval was not required since this study is a meta‐synthesis.

## Conflicts of Interest

The authors declare no conflicts of interest.

## Protocol Registration

This meta‐synthesis was registered with PROSPERO (Protocol No. CRD42022298218).

## No Patient or Public Contribution

This meta‐synthesis requires no direct participation of patients.

## Supporting information


Data S1.

Data S2.

Data S3.

## Data Availability

The data that support the findings of this study are available from the corresponding author upon reasonable request.
