# Supplementary material for: Substance Use Disorders in Adolescents: A Qualitative Systematic Review
Source: J Adv Nurs. 2025 Apr 19;82(3):2084–100. doi: 10.1111/jan.16967 (PMC12907611; doi:10.1111/jan.16967)
Supplement: Supplementary file 3 — Data S3. [file JAN-82-2084-s003.docx]

| **Supplementary File 3. Search Strategy** |
| --- |
| **PUB MED** |
| ("substance-related disorders"[All Fields] OR "chemical dependence"[All Fields] OR "drug abuse"[All Fields] OR "drug addiction"[All Fields] OR "drug dependence"[All Fields] OR "drug habituation"[All Fields] OR "drug use disorder"[All Fields] OR "drug use disorders"[All Fields] OR "prescription drug abuse"[All Fields] OR "substance abuse"[All Fields] OR "substance addiction"[All Fields] OR "substance dependence"[All Fields] OR "substance use"[All Fields] OR "substance use disorder"[All Fields]) AND ("adolescents"[All Fields] OR "adolescence"[All Fields] OR "teenagers"[All Fields] OR "teens"[All Fields] OR "youth"[All Fields]) AND ("life experience"[All Fields] OR "perception"[All Fields]) AND ("qualitative research"[All Fields] OR "qualitative study"[All Fields] OR "descriptive qualitative"[All Fields]) |
| **EBSCO CINAHL COMPLETE** |
| AB ( “Substance Related Disorders” OR “Chemical Dependence” OR “Drug Abuse” OR “Drug Addiction” OR “Drug Dependence” OR “Drug Habituation” OR “Drug Use Disorder” OR “Drug Use Disorders” OR “Prescription Drug Abuse” OR “Substance Abuse” OR “Substance Addiction” OR “Substance Dependence” OR “Substance Related Disorder” OR “Substance Use” OR “Substance Use Disorder” ) AND AB ( “Adolescents” OR “adolescence” OR “Teenagers” OR “Teens” OR “Youth” ) AND AB ( “Life Experience” OR “Perception” ) AND AB ( “qualitative research” OR “qualitative study” OR “descriptive qualitative” ) |
| **WEB OF SCIENCE** |
| (((AB=(“substance-related disorders” OR “chemical dependence” OR “drug abuse” OR “drug addiction” OR “drug dependence” OR “drug habituation” OR “drug use disorder” OR “drug use disorders” OR “prescription drug abuse” OR “substance abuse” OR “substance addiction” OR “substance dependence” OR “substance use” OR “substance use disorder”)) AND AB=(“adolescents” OR “adolescence” OR “teenagers” OR “teens” OR “youth”)) AND AB=(“life experience” OR “perception”)) AND AB=(“qualitative research” OR “qualitative study” OR “descriptive qualitative”) |
| **SCIENCE DIRECT** |
| ("substance-related disorders" OR "drug abuse" OR "substance addiction" OR "substance use" OR "substance use disorder") AND ("Adolescents") AND ("life experience OR perception") AND ("qualitative research") Filtered Research article and english article |
| **SCOPUS** |
| TITLE-ABS-KEY ( "substance-related disorders" OR "chemical dependence" OR "drug abuse" OR "drug addiction" OR "drug dependence" OR "drug habituation" OR "drug use disorder" OR "drug use disorders" OR "prescription drug abuse" OR "substance abuse" OR "substance addiction" OR "substance dependence" OR "substance use" OR "substance use disorder" ) AND TITLE-ABS-KEY ( "adolescents" OR "adolescence" OR "teenagers" OR "teens" OR "youth" ) AND TITLE-ABS-KEY ( "life experience" OR "perception" ) AND TITLE-ABS-KEY ( "qualitative research" OR "qualitative study" OR "descriptive qualitative" ) |
